# Mortality and Morbidity of Fireworks-Related Burns on the Annual Last Wednesday of the Year Festival (Charshanbeh Soori) in Iran: An 11-Year Study

**DOI:** 10.5812/traumamon.11700

**Published:** 2013-08-14

**Authors:** Reza Vaghardoost, Yaser Ghavami, Behnam Sobouti, Mohammad Reza Mobayen

**Affiliations:** 1 Burn Research Center, Shahid Motahari Hospital, Tehran University of Medical Sciences, Tehran, IR Iran; 2 Department of Pediatric Infectious Diseases, Shahid Motahari Hospital, Tehran University of Medical Sciences, Tehran, IR Iran

**Keywords:** Costs and Cost Analysis, Morbidity, Burns

## Abstract

**Background:**

Management of firework-related injuries is costly for the patient, society, and government.

**Objectives:**

Evaluating effective factors yielding to such injuries may lead to better management of patients and decreased costs and morbidities.

**Patients and Materials:**

This retrospective cross-sectional study was performed on burn patients referred to Shahid Motahari Burns Hospital on Charshanbeh Soori day festival during the period extending from March 2000 to March 2011 (11 days in an 11-year period). Demographic data, causes of burn injury, severity, and affected body parts were recorded. Data were analyzed using SPSS version 16.

**Results:**

There were164 patients in the study with a mean age of 18.34 ± 9.31 years; 87% (145/164) were male. Homemade grenades were the most frequent cause of injury. Hand injury was reported in 56% (92/164) of the cases. Amputation was executed in 7 (4.3%) cases, and 6 (3.7%) patients died due to severe burn injuries and facial damage.

**Conclusions:**

Fireworks- related injuries during Charshanbeh Soori ceremony causes significant morbidities and damage to different body parts (especially upper limbs and face), and some of these injuries will lead to life time disabilities, amputations, and even death. As most of the injured patients are young teenagers and children, special consideration must be taken into account to prevent long term morbidities.

## 1. Background

Festivals and celebrations are held in most countries worldwide each year. In these special occasions, people usually use fireworks (FW) for entertainment and enjoyment which sometimes may lead to a series of unfortunate events. There have been numerous reports and studies on FW-related injuries ( 1 - 4 ). The last Wednesday of the Persian year is known as “Charshanbeh Soori” and is celebrated each year. It is a traditional festival which dates back to ancient times and is held at the last Tuesday night of the year (based on the Persian calendar). During previous centuries, this celebration was mostly peaceful and only a simple bonfire was lit during festivals. However, nowadays advent of new firework devices and dangerous hand-made grenades has changed this calm festival into a disastrous event (Table 1). Reports indicate that too many victims, especially children and young adults are injured by various types of FW. Instead of celebrating New Years Eve aside their families, they spend their time in hospitals suffering pain and disabilities. Furthermore, the injuries of many of these victims lead to permanent disabilities (i.e. amputation or blindness), resulting in life-long problems ( 5 - 8 ).

**Table 1.** Definition of Fireworks Types

Types of Fireworks	Definition
**Homemade grenade**	A homemade small shell containing an explosive, which is thrown by hand to make an explosion and loud sound.
**Sparklers**	Hand-held fireworks that burn slowly and give off a shower of colored sparks from the burning tip.
**Black powder**	A mixture of sulfur, charcoal, and potassium nitrate where the sulfur and charcoal work as fuel.
**Rockets and missiles**	Bottle rockets with a long stick rise quickly into the sky and explode with crackles or stars.
**Fountains**	Fireworks that expel a multitude of sparks as their effect. They are usually safe.
**Ground spinners**	These spin around randomly at ground level, shooting out colored sparks and flames.
**Fuse detonated noise makers**	Small explosives that produce a loud noise.
**Gas capsule**	A mini capsule filled with natural gas with a fuse that is thrown on the floor and explodes.

## 2. Objectives

The aim of this study was to evaluate the prevalence of burn injuries and related factors following the Persian last Wednesday of the year festival (Charshanbeh Soori).

## 3. Patients and Methods

This retrospective cross-sectional study was performed on patients who were admitted to Shahid Motahari Burns Hospital on the last Wednesday of the year Festival (Charshanbeh Soori) during the period extending from March 2000 to March 2011 (11 year period). Data on the number of injured patients, age, sex, and socio-economic status of the patients, and also the site, type, severity, and outcome of the burn injury, and the type of fireworks involved were collected (Table 2). The types of fireworks causing injury were extracted from the patients’ medical records or obtained verbally directly from the patients or companions and were classified.

**Table 2.** Demographic Data of Injured Patients at Persian Wednesday Eve Festival

Variable	No. (%)
**Age, mean ± SD**	18.34± 9.31
**Gender**	
Male	145 (87)
Female	19 (13)
**Education**	
Undergraduate	121 (73.8)
Diploma	32 (19.5)
BS	8 (4.88)
PhD	3 (1.82)
**Employment**	
Non employed	143 (87.2)
Employed	21 (12.8)
**Using Status**	
User	102 (62.2)
Producer	24 (14.6)
Bystander	38 (23.2)
**Place**	
Street	131 (79.9)
Home	17 (10.4)
School	16 (9.7)
**Injury**	
Deep second degree burn	98 (59.8)
Third degree burn	34 (20.7)
Limb amputation	7 (4.3)
Wound	6 (3.7)
Multiple injuries	16 (9.8)
Death	6 (3.7)

In recent years due to stringent laws for using or producing fireworks, these devices usually are made illegally at home which implies that they may be produced under unacceptable standards.

Data were analyzed using SPSS for Windows (version 16). Frequency distributions were calculated for each variable and descriptive statistics was used to summarize the data. For continuous variables, mean (SD) was calculated. Categorical variables were summarized as percentages and assessed using Chi-2 or Fisher’s exact tests.

## 4. Results

**Figure 1.**
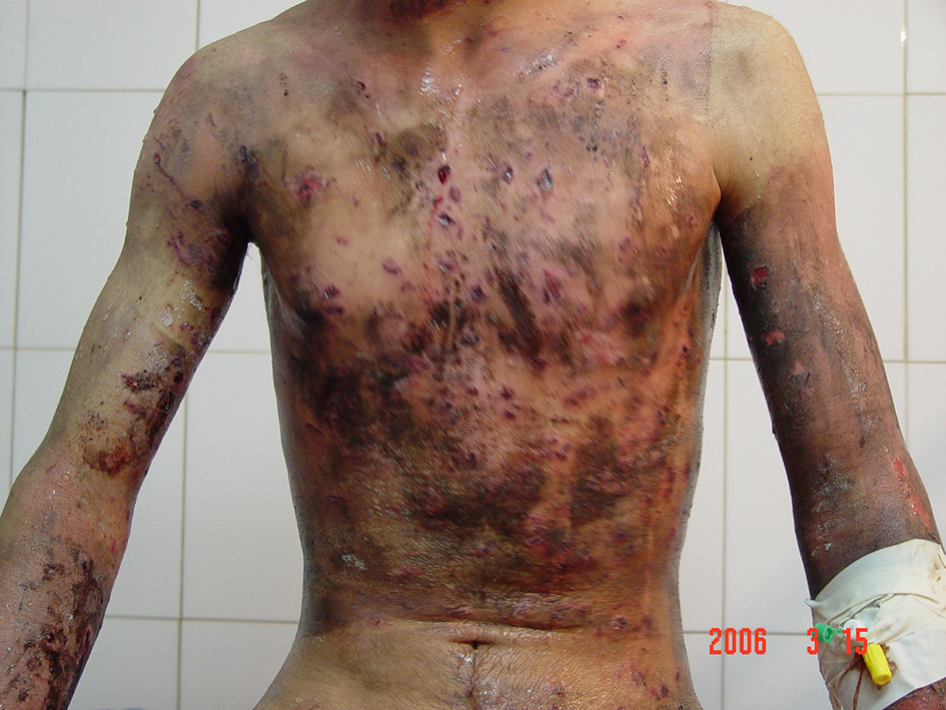
Devastating Effect of Fireworks on a Patient (Trunk and Limb Injuries)

**Figure 2.**
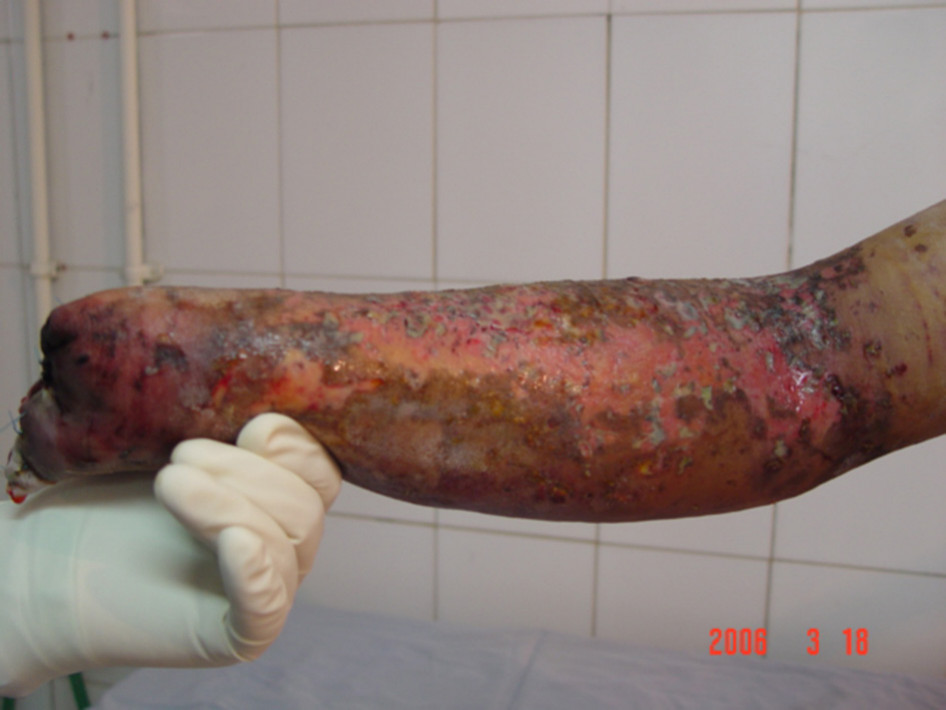
Amputation Due to Fireworks Injury

**Figure 3.**
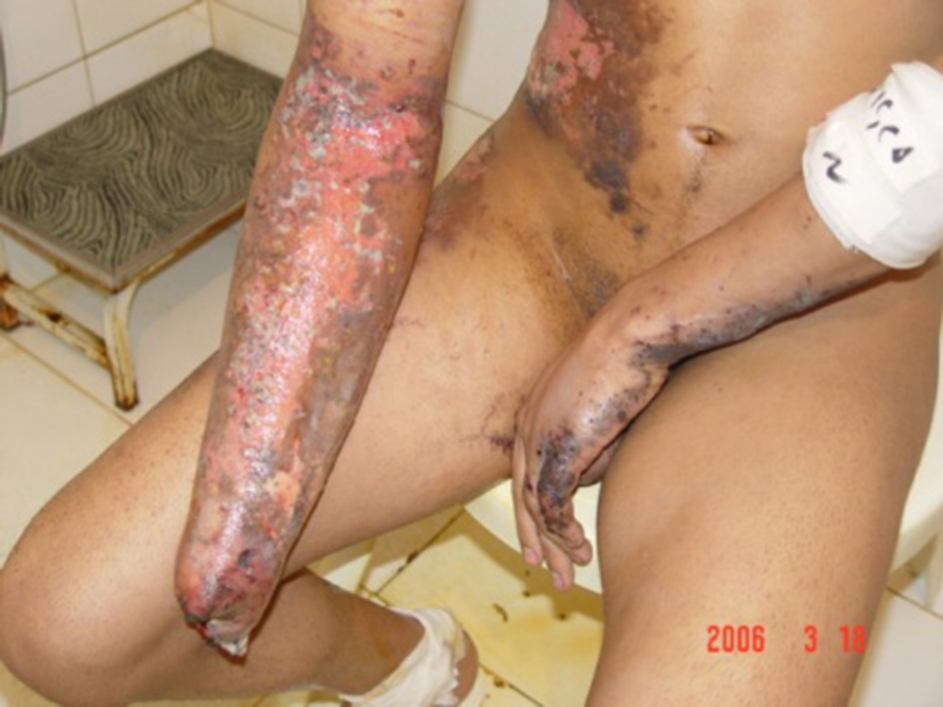
Hand Injury Due to Fireworks

In this study 164 patients hospitalized due to fireworks injuries were assessed with a mean age of 18.34 ± 9.31 years. The majority of injured patients were admitted on the last Tuesday night of the year and the following day (Wednesday). Eighty seven percent (145/164) of the patients were male(Figure 1). Homemade grenades were the most frequent cause of injuries (Table 3). Hand injury was reported in 56% of the cases (Figure 2). Amputation was executed in 7 cases (Figure 3); 6 patients died due to severe burn injuries and facial damage. The majority of the injuries occurred in the 15-20 year-old (29.4%) age group (Table 3). In the entire group of study patients, 75% were fireworks users and 3% were producers; the remaining 22% of injured patients were bystanders watching the ceremony. The majority of the injuries occurred on the street (74.5%), whereas fewer injuries occurred at home (23.4%) or at school (2.1%).

**Table 3.** Relation between Site of Injury and Type of Firework Used

	HMG^a^	Sparkles	Black Powder	R&M	Fountain	Ground Spinner	FDNM	Gas Capsule	Total
**Hand**	56	19	6	1	3	-	2	5	92
**Face**	3	2	4	-	-	-	6	4	19
**Foot**	16	-	-	2	-	7	-	4	29
**Eye**	3	2	4	-	-	-	3	2	14
**Trunk**	5	-	-	7	-	-	-	4	16
**Multiple injuries**	7	-	-	-	-	-	-	4	11

^a^Abbreviations: HMG, homemade grenade; R&M, rockets and missiles; FDNM, fuse detonated noise maker

## 5. Discussion

Use of dangerous types of fireworks and improper application of these materials are the main causes attributing to injuries in festivals and ceremonies held throughout the world. In the present study, 164 individuals suffered from firework-related injuries; 79% of them were males with a mean age of 21 years. The majority of the injuries occurred in the 15-20 years age group (mean age: 18.34 ± 9.31). These results are in accordance with the findings of other studies that have reported males as the high-risk group for such injuries (1, 9, 10). In some studies fireworks-related injuries were more prevalent among those aged 18 years and younger; also teenagers are at the highest risk for these injuries (9, 11, 12). In a study by Tavakoli et al. on 197 patients relevant to Charshanbeh Soori, found the majority of patients to be males, and FDNM and HMG to be the most frequent firework devices causing injury. Also hand injury constituted a high percentage rate (39.8%) (8). In a 10-year study in India, the majority of the injured cases were children 5-14 years old, and misuse of the devices was reported as the main cause of these injuries (9). In the present study, homemade grenades were the most frequent type of fireworks causing injury followed by rockets and missiles. However in a study by Tavakoli et al., it was noted that FDNM were the most frequent used fireworks causing injury (8). A 10-year study in India by Puri et al. indicated that fountains and flares were responsible for the majority of injuries and also that misuse was the most common reason for injury (9). This may be due to increasing amounts of illegally made fireworks devices (especially homemade grenades) after institution of sanctions against using fireworks and also lack of awareness among users. In a 5-year study by Vassilia et al. in Greece, fireworks related injuries were common in males and limb injuries were the most common sites affected (10). A study by Morell et al., they found flying objects and foreign bodies to be the most common devices causing injuries; he showed that direct heat (51%), flying objects (33%), and foreign bodies penetrating the eyes (10%) as the most prevalent mechanisms of injury in fireworks injured cases. They also mentioned that affected girls were mainly innocent bystanders (13). On the other hand, whistles were the main type of firework causing injury in Sheller’s research in Denmark (14).In our study, bystanders (38/164) who suffered injuries were mostly boys (17/164), and most of the injuries occurred across the streets. Morell et al. noted that parks and streets were the main scenes of accidents (13). It is worthy to note that determining the location of accidents is important, because supervision at the main sites can significantly reduce the danger extent of that event, and in the case of fire, rescue forces can easily access the site of accident. In a study by Tavakoli et al. they found that burns, lacerations, contusions, and foreign bodies were the main types of injury; also extremities were likely sites of injury by fireworks (8). Puri et al. reported that the hands to be the main site of injury in 80% of the cases they studied (9). In other studies in Australia, Saudi Arabia, England, India, Ireland, and Denmark, reports indicate that hands are the main site of injury also (4, 5, 9, 11, 14, 15). In our study also the hands were the most common site of injury. Puri reported that holding fireworks (especially fountains) in the hand and unexpected explosion caused most of the injuries (9). In our study, holding HMGs in hands and unexpected explosions were the most common causes of hand injuries. In several other studies, eyes were reported as the main body part injured by fireworks. For example, Vassilia et al. reported eye trauma as the most frequent type of injury in children injured by fireworks which in some cases led to blindness (10). In another study by Zohar et al., eye and face injuries were reported in 24% of cases. In the present study, eye injuries occurred in 14 cases. This low amount is due to the fact that most of the eye injured patients were transported or referred to hospitals which had an ophthalmology department. Educational level and employment status of patients is another factor which had significant statistical correlation with injuries. Injuries occurred mostly in undergraduate and unemployed individuals (P < 0.001). In this study, gas capsule was the most dangerous device, and its usage led to death in 4 patients due to its massive explosive effect and fatal injuries to vital organs. Our findings indicate that the amount of burn injuries, morbidity, and mortality rates have decreased during recent years. Social programs, educational programs for children, and participation of national media (television, radio, and newspapers) informing the public society, has dramatically increased the awareness of individuals about dangers and complications of using illegal and inappropriate fireworks. It is obvious that education can play a significant role in reducing casualties. Our results indicate that the majority of the injuries causing complications was due to using HMGs (which is an illegal fireworks device) and was most common in the 15-20-year age group. It is obvious that institution of sanctions and preventive laws can impede further damages. However prevention without education is not sufficient. The sources of initial ingredients for making homemade devices should be investigated and barred. Also supervision of parents and accompanying them during these festivals can be a beneficial in preventing hazardous effects of using fireworks. This study was on patients with fireworks-related burn injuries who were admitted to a major burns center in Tehran. This study did not include those who were admitted to ophthalmology centers due to firework-related eye injuries or patients who were admitted to major trauma centers or general hospitals due to multiple traumas. Moreover, out-patients were not included in this study. Further studies are needed to study all individuals injured due to fireworks, as a considerable difference in the severity of injury may exist.

Fireworks-related injuries during Charshanbeh Soori ceremony causes significant morbidities and damage to different body parts (especially upper limbs and face), and some of these injuries will lead to life-long disabilities, amputations, and even death. As most of the injured patients are young adults and children, special consideration must be taken into account to prevent long term associated morbidities.
